# Endometrial Collagen in Woolly Monkey (*Lagothrix l. poeppiggi*) and the Uakari ( *Cacajao calvus* )

**DOI:** 10.1111/jmp.70055

**Published:** 2026-01-11

**Authors:** Thyago Habner de Souza Pereira, Frederico Ozanan Barros Monteiro, Francisco Antônio Félix Xavier Júnior, Isadora Oliveira de Carvalho, Janaina Serra Azul Monteiro Evangelista, Ana Kelen Felipe Lima, Pedro Mayor

**Affiliations:** ^1^ Universidade Federal Rural da Amazônia (UFRA), Programa de Pós‐graduação em Saúde e Produção Animal na Amazônia Belém Pará Brazil; ^2^ Universidade Estadual do Ceará (UECE), Programa de Pós‐graduação em Ciência Animal Fortaleza Ceará Brazil; ^3^ Universidade Estadual do Ceará (UECE), Pós‐graduação em Ciências Fisiológicas Fortaleza Ceará Brazil; ^4^ Universidade Federal do Norte do Tocantins (UFNT), Programa de Pós‐Graduação em Sanidade Animal e Saúde Pública nos Trópicos Araguaína Tocantins Brazil; ^5^ Departament de Sanitat i d’Anatomia Animals Facultat de Veterinària, Universitat Autònoma de Barcelona (UAB) Barcelona Spain; ^6^ ComFauna, Comunidad de Manejo de Fauna Silvestre en la Amazonía y en Latinoamérica Iquitos Peru; ^7^ Museo de Culturas Indígenas Amazónicas Iquitos Loreto Peru

**Keywords:** endometrial architecture, neotropical primates, picrosirius red staining, polarized light microscopy, reproductive cycle phases, type I collagen, type III collagen

## Abstract

**Introduction:**

This study microscopically characterizes collagen changes and their relationship with endometrial architecture in two Neotropical primates.

**Methods:**

Uterine fragments from six *Lagothrix l. poeppigii* and six *Cacajao calvus* in different reproductive phases were histologically processed using Picrosirius Red staining.

**Results:**

In the functional layer, collagen was highest in the early proliferative phase (5.19% ± 2.53%; *p* < 0.001), whereas the basal layer had the lowest percentage (5.85% ± 4.69%; *p* < 0.01). Type I collagen predominated, while type III was less expressed. Type I collagen varied significantly between proliferative phases (*p* < 0.001). The basal layer exhibited more fibers in the secretory phase (14.03% ± 8.50%; *p* < 0.001). Type III collagen peaked in the functional layer (0.41% ± 0.17%; *p* < 0.001) and basal layer (0.69% ± 0.46%; *p* < 0.05).

**Conclusion:**

Picrosirius Red staining with polarized light effectively highlights collagen organization, aiding reproductive phase characterization in Neotropical primates.

## 1 Introduction

The endometrium exhibits dynamic changes in the extracellular matrix (ECM) composition and architecture during homeostasis, gestation, and in response to endometrial‐associated pathologies [1, 2]. This matrix promotes regeneration and maintenance throughout the uterine cycle, and it is composed of a complex network of proteins, such as fibronectin, proteoglycans, and collagen, the most abundant macromolecule in the ECM [3, 4].

Previous studies have identified several types of collagens associated with the ECM in the endometrium and decidua, whose expression changes throughout the menstrual cycle or in response to steroidal sex hormones [5, 6]. which are essential for reproductive function, adapting the functional layer of the endometrium [7]. However, despite the few studies conducted in humans or rodents [7], the role of the ECM in epithelial and estrous cell function is still not well understood.

Collagen expression in the ECM may provide important information on the dynamics of uterine tissue remodeling in species with menstrual cycles [5, 7]. These changes in the composition and organization of the ECM can be identified by immunohistochemistry (IHC), such as the use of picrosirius red (F3Ba) used in the identification and quantification of collagen [8, 9, 10] during the uterine remodeling process [11, 12, 13]. Type I collagen is the main fibrillar collagen in the endometrium of women and provides the greatest strength and is involved in the regulation of cell proliferation, migration, and differentiation of mesenchymal stem cells. Type III collagen is also found in the regulation of tissue regeneration, although its specific role is still not understood [14].

Although non‐human primates (NHP) are widely used as biological models for understanding reproductive morphology and evolutionary adaptations [15], studies on Neotropical species remain scarce, particularly for the woolly monkey (*Lagothrix l. poeppigii*) and the uakari ( *Cacajao calvus* ), both non‐menstruating and currently threatened species [16]. Nevertheless, documenting the uterine structure of these species is of great scientific value, as such data are virtually unavailable and may represent the only record for these taxa.

In this context, the present study aims to microscopically characterize the endometrium of *L. l. poeppigii* and *C. calvus* , describing its structural organization and quantifying type I and III collagen across reproductive phases using F3Ba staining. This descriptive approach contributes to the limited knowledge of uterine ECM composition in New World primates and provides a comparative framework for understanding reproductive adaptations in non‐menstruating species.

## 2 Materials and Methods

### 2.1 Study Area

This study was conducted in the Yagua indigenous community of Nueva Esperanza, located in the Yavarí‐Mirín River basin (S 04°19.53; W 71°57.33; UT5: 00), a geographically isolated and well‐preserved forest along the border between Brazil and Peru in the Northeastern Peruvian Amazon. The study areas span over 107 000 ha of continuous forest with a predominance of non‐flooding upland. The region's climate is equatorial, with an annual temperature ranging from 22°C to 36°C, relative humidity between 80% and 100%, and annual precipitation from 1500 to 3000 mm. The seasons are defined in dry periods (January–February and July–September) and rainy periods (March–June and October–December).

### 2.2 Collection of Biological Material and Histological Processing

This research was approved by the Research Ethics Committee for Experimentation in Wildlife at the Dirección General de Flora y Fauna Silvestre from Peru (License 0229‐2011‐DGFFS‐DGEFFS). The samples were sent to the Universidade Federal Rural da Amazônia (UFRA) and to the Universidade Estadual do Ceará (UECE) using the CITES/IBAMA export license (No. 14BR015991/DF) and the research was authorized by the Ethics Committee for the use of UFRA animals (CEUA/UFRA protocol 008/2016).

Between 2010 and 2014, hunters living in the YMR were trained to collect the abdominal and pelvic organs of all hunted preys and preserve the samples in a 4% buffered formaldehyde solution (v/v) until histological processing [15, 17]. This activity was included within an ongoing participatory conservation program that involves local hunters in implementing community‐based wildlife management. From the collected biological samples, we selected the genital organs from 12 non‐pregnant female primates, including six woolly monkeys (*L. l. poeppigii*) and six red uakaris ( *C. calvus* ). The six non‐pregnant females of both species were classified according to endometrial performance in early proliferative (*n* = 2), proliferative (*n* = 2), and secretory uterine phase (*n* = 2) [15]. This methodology assured that no animal was killed other than those harvested as part of local hunters' normal activities, and hunters were not paid. A postmortem examination was conducted to verify that none of the individuals died due to any reproductive disorder.

### 2.3 Endometrial Collagen Analysis and Quantification

Biological samples were dehydrated in increasing concentrations of ethanol, subjected to clearing in xylol and subsequently infiltrated and embedded in paraffin. Microtomy was performed in 3 μm thickness and stained with Hematoxylin–Eosin (H&E) and Masson's trichrome. H&E staining is the standard staining technique and was used to assess the quality of biological material and tissue architecture, while Masson's trichrome allows the identification of collagen in blue and regions of fibrin in red. Sections were examined under a light microscope at total magnification of 100×, 400×, and 1000×.

**FIGURE 1:**
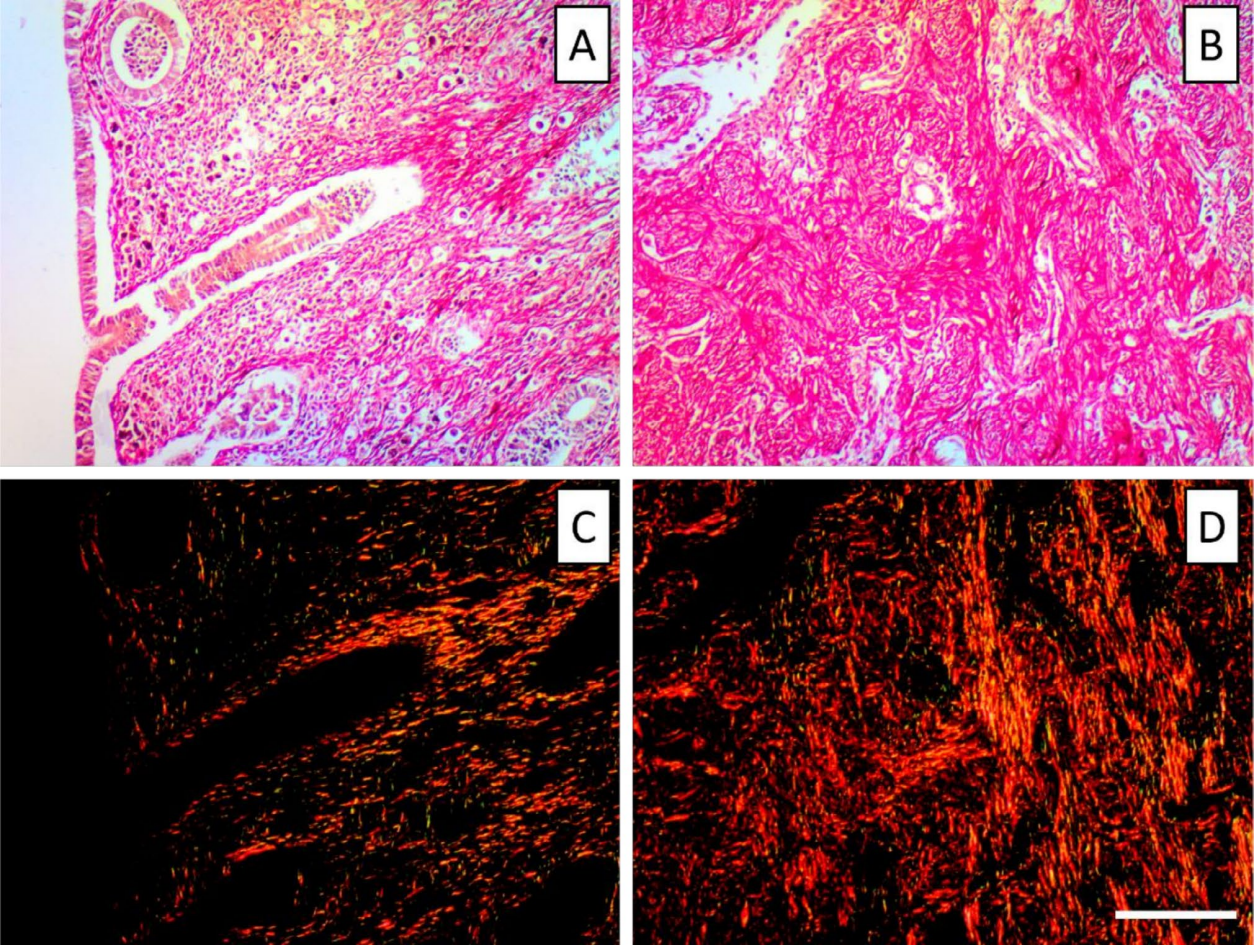
Histological sections of the uterus of *Cacajao calvus* during the early proliferative phase, stained with Picrosirius Red and examined under non‐polarized (A, B) and circularly polarized light (C, D). In non‐polarized images (A, B), collagen‐rich regions appear uniformly red, allowing visualization of general stromal architecture. Under polarized light (C, D), birefringence highlights collagen fiber organization, with type I collagen exhibiting red–orange tones and type III collagen appearing in greenish hues. Panels A and C depict the functional layer, characterized by a more loosely arranged stroma and early glandular development, whereas panels B and D illustrate the basal layer, where collagen fibers are denser and more compact. Magnification: 200×. Nikon Eclipse Trinocular/NIS‐Elements 4.0. Scale bar: 0.25 mm.

Uterine fragments were subsequently stained with Picrosirius Red (F3Ba) to identify collagen fiber types I and III in the studied uterine phases. Quantitative analysis of endometrial collagen was conducted according to the modified technique by Morais et al. [18] and de Moura et al. [19]. Samples stained with Picrosirius Red were examined under both polarized and unpolarized light. Figure 1 presents photomicrographs of the *C. calvus* uterus in the early proliferative phase, highlighting structural differences between non‐polarized and circularly polarized imaging. Under polarized light microscopy, collagen birefringence can be observed in the reddish‐yellow color, being attributed to type I collagen, while the greenish tones are associated with type III collagen [9, 20]. Random fields from the functional (*n* = 5) and basal (*n* = 5) layers of the endometrium from each uterine specimen were captured with and without circular polarization (Nikon Eclipse Cipol microscope with Nikon DS‐Ri2 digital camera), and digital images of the histological sections were obtained in a standardized way (total magnification of 200×).

**FIGURE 2:**
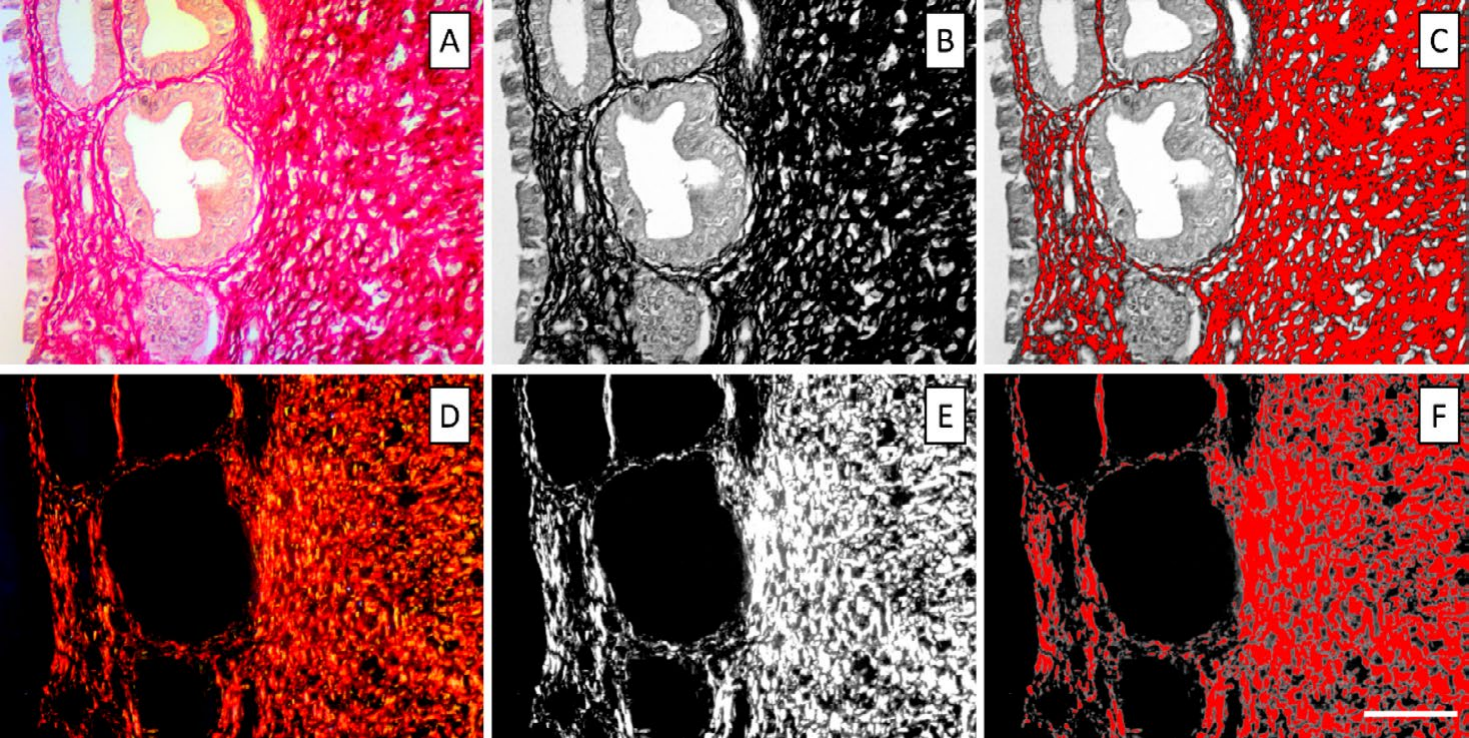
Histological sections of the endometrium of *Cacajao calvus* during the secretory phase, stained with Picrosirius Red and examined under non‐polarized and polarized light. (A) Functional layer of the endometrium visualized under non‐polarized light, showing stromal architecture and collagen distribution with conventional birefringence not yet enhanced. (D) The same region observed under circularly polarized light, highlighting birefringent collagen fibers, where reddish–yellow tones correspond primarily to type I collagen and greenish hues indicate type III collagen. (B, E) Binarized versions of the images used to isolate collagen‐positive regions following image thresholding. (C, F) Digital segmentation of collagen‐expressing areas (in red) used for quantitative analysis in non‐polarized (C) and polarized (F) sections. All images represent the functional layer during the secretory phase. Magnification: 200×. Microscope: Nikon Eclipse (trinocular) with NIS‐Elements 4.0 software. Scale bar = 0.25 mm.

**FIGURE 3:**
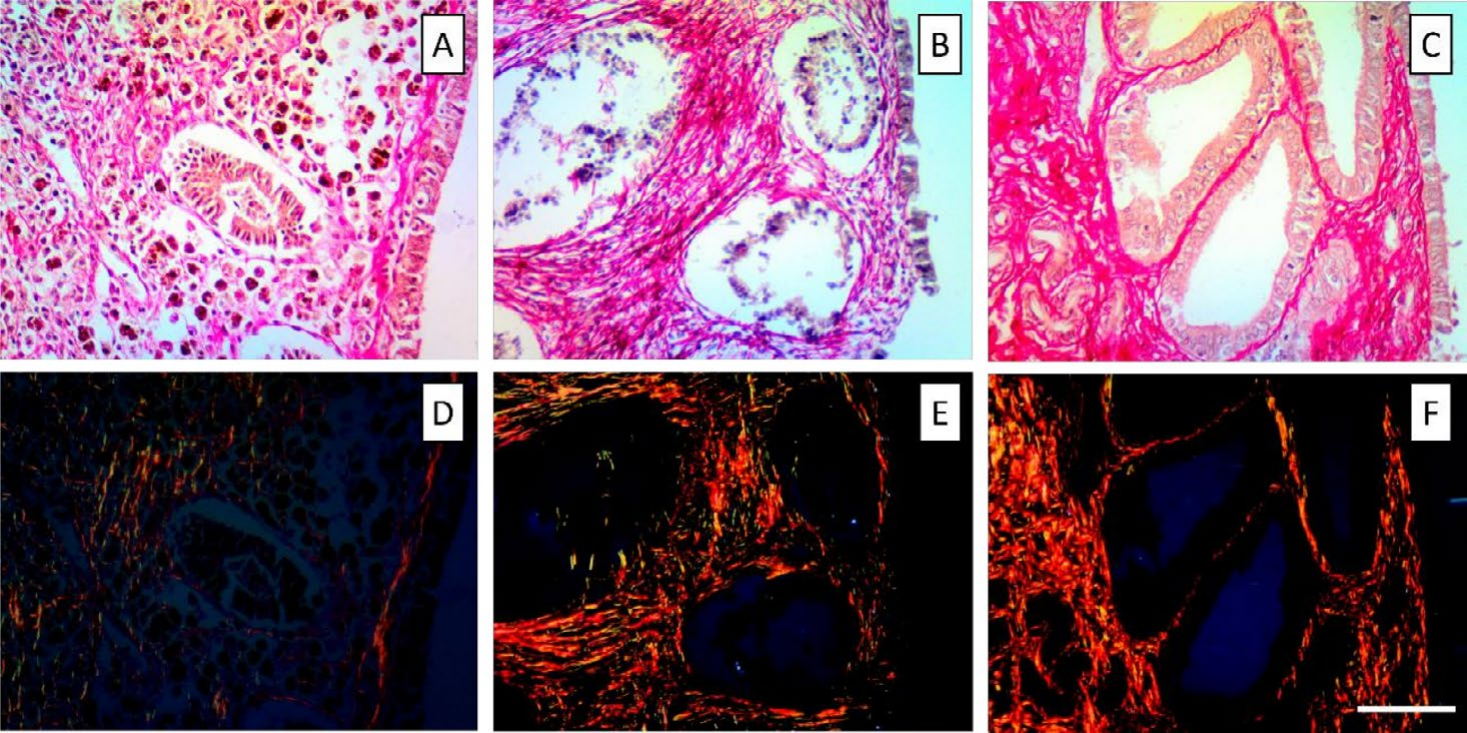
Histological sections of the endometrium of *Cacajao calvus* illustrating collagen organization across the reproductive cycle under non‐polarized and circularly polarized light. (A, D) Early proliferative phase: The functional layer is thin, with sparse stromal collagen; birefringent type I fibers (reddish yellow) and type III fibers (greenish) become distinguishable under polarized light (D). (B, E) Late proliferative phase: Progressive thickening of the functional layer with increased glandular development; collagen fibers exhibit more organized distribution, with enhanced birefringence in polarized images (E). (C, F) Secretory phase: The endometrium displays a widened stromal compartment and enhanced collagen deposition, particularly in the basal layer; polarized light (F) highlights the predominance of type I collagen. Images were obtained at 200× magnification using a Nikon Eclipse trinocular microscope with NIS‐Elements 4.0. Scale bar = 0.25 mm.

The differences in collagen fiber organization between the reproductive phases are clearly demonstrated in the photomicrographs, where the functional and basal layers exhibit distinct patterns of collagen deposition and polarization (Figures 2 and 3). All collagen quantification was therefore performed exclusively on images obtained under polarized light. To quantify these variations, a morphometric analysis of tissue sections was conducted using ImageJ software, version 1.8.0. The software was calibrated with the Gray Scale, background lighting was removed, and images were adjusted to delineate the functional and basal layers of the endometrium. All images were converted to 8‐bit grayscale, and total collagen was quantified using the Threshold tool. To differentiate collagen types I and III, the Split Channels tool was employed to separate the RGB color components, followed by quantification using the red and green channels for collagen types I and III, respectively.

Collagen was quantified in each field analyzed and the values were expressed as a percentage of the area. Finally, we calculated the average percentage based on the five random fields in each uterine specimen.

### 2.4 Statistical Analysis

All evaluated parameters were tested for normal distribution using the Shapiro–Wilk test. One‐way ANOVA tests were performed to evaluate the difference in the proportion of collagen type I and III between the different uterine phases (early proliferative, final proliferative, and secretory), considering the parametric data. Means between groups were compared using Turkey's multiple comparisons test. Statistical analysis and graphics were performed using the GraphPad Prism 8.0.1 software. Differences with a probability value of 0.05 or lower were considered significant. Values are presented as mean ± SD.

## 3 Results

**FIGURE 4:**
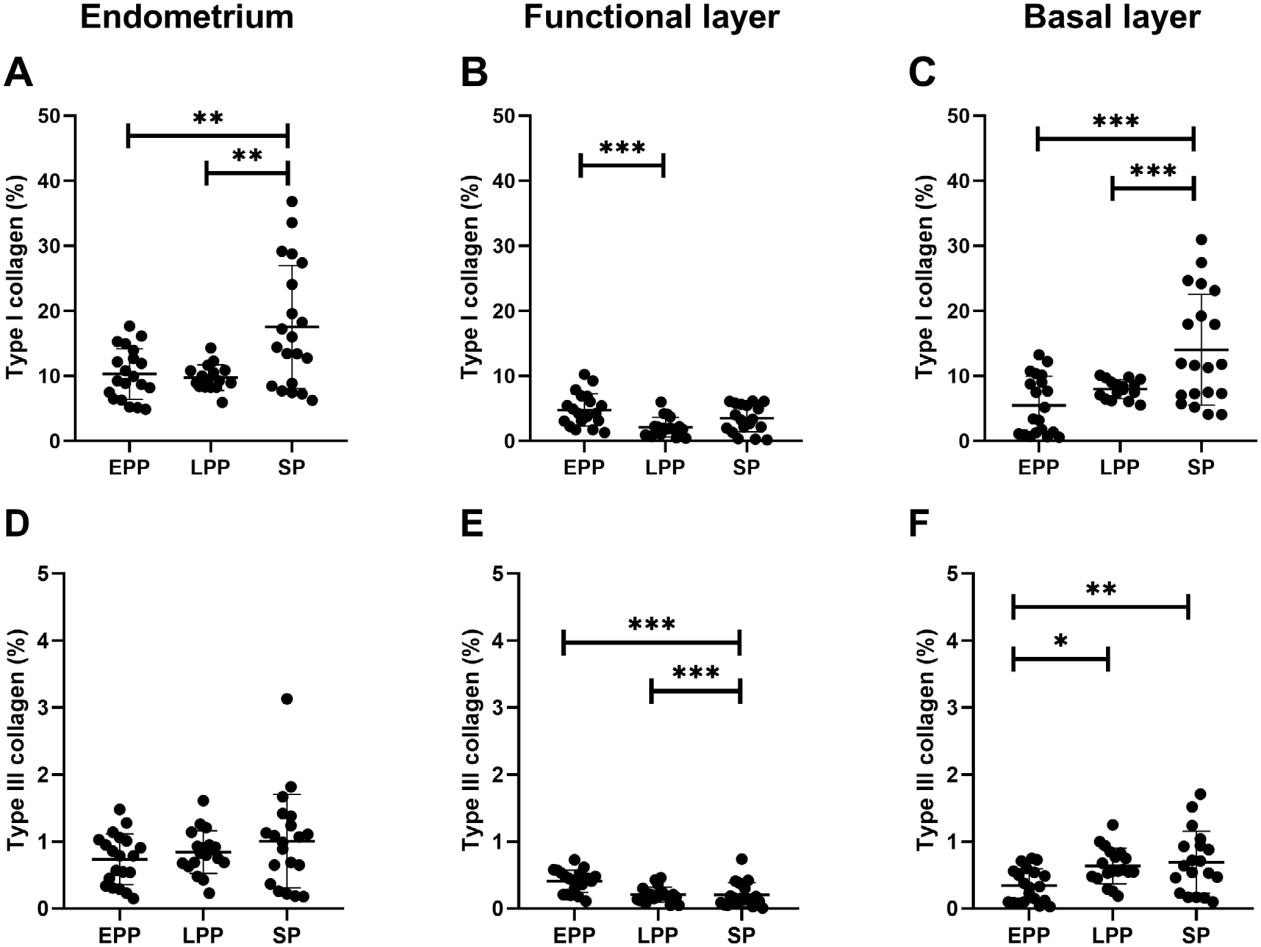
Quantitative evaluation of collagen expression in the endometrium of *Lagothrix l. poeppigii* and *Cacajao calvus* throughout the reproductive cycle, with emphasis on type I (A–C) and type III (D–F) collagen fibers. The overall percentage of collagen (A, D), in the functional (B, E) and basal (C, F) layers is observed. EPP: Early proliferative phase; LPP: Late proliferative phase; SP: Secretory phase; * < 0.05; ** < 0.01; *** < 0.001.

The mean percentage of collagen in the functional and basal layers of the endometrium is shown in Table 1. The overall percentage of endometrial collagen of *L. l. poeppigii* and *C. calvus* showed significant difference between proliferative and secretory phases (*p* = 0.002; Figure 4).

**TABLE 1:** Mean ± SD and range [minimum maximum] of the proportion of collagen observed in the functional, basal layers of the endometrium of the *Cacajao calvus* (*n* = 6) and *Lagothrix poeppigii* (*n* = 6) in different phases of uterine cycle.

Type collagen	Layers	Early proliferative	Late proliferative	Secretory
Type I	Functional layer (%)	4.79 ± 2.50^a^ [1.33–10.24]	2.15 ± 1.50^b^ [0.44–6.03]	3.52 ± 2.10 [0.20–6.17]
	Basal layer (%)	5.50 ± 4.46^b^ [0.59–13.25]	7.99 ± 1.46^b^ [5.51–10.10]	14.03 ± 8.50^a^ [4.09–30.98]
	Geral (%)	10.31 ± 3.91^b^ [4.89–17.65]	9.77 ± 1.97^b^ [5.96–14.31]	17.55 ± 9.44^a^ [6.28–36.82]
Type III	Functional layer (%)	0.41 ± 0.17^a^ [0.11–0.73]	0.21 ± 0.11^b^ [0.05–0.46]	0.21 ± 0.18^b^ [0.01–0.74]
	Basal layer (%)	0.34 ± 0.25 [0.03–0.75]	0.64 ± 0.27 [0.19–1.25]	0.69 ± 0.46 [0.10–1.71]
	Geral (%)	0.74 ± 0.38^b^ [0.15–1.48]	0.84 ± 0.32^a^ [0.23–1.61]	1.01 ± 0.70^a^ [0.18–3.13]

*Note:* Different letters on the same line indicate statistically significant difference (*p* < 0.05).

The percentage of collagen in the functional layer was 5.19% ± 2.53% in the early proliferative, 2.34% ± 1.55% in the late proliferative and 3.72% ± 2.22% in the secretory phase. We observed significant differences between the early and final proliferative periods (*p* < 0.001). Regarding the basal layer, the overall percentage was 5.85% ± 4.69%, 8.60% ± 1.50% and 14.83% ± 8.99% in the early proliferative, late proliferative and secretory phase, in this order. Significant differences were observed between early and final proliferative with secretory phase (*p* < 0.001 and *p* = 0.01, respectively).

The overall percentage of type I collagen was higher in the secretory phase with 17.55% ± 9.44%. A significant difference was observed between the early proliferative and secretory periods (*p* = 0.002) and late proliferative with secretory (*p* = 0.001). In the functional layer, there was greater expression in the early proliferative phase with 4.79% ± 2.50% when compared to the final period with 2.15% ± 1.50% (*p* < 0.001). We also observed a significant difference in the basal layer between the secretory and the early (*p* < 0.001) and the final proliferative phases (*p* = 0.01).

**FIGURE 5:**
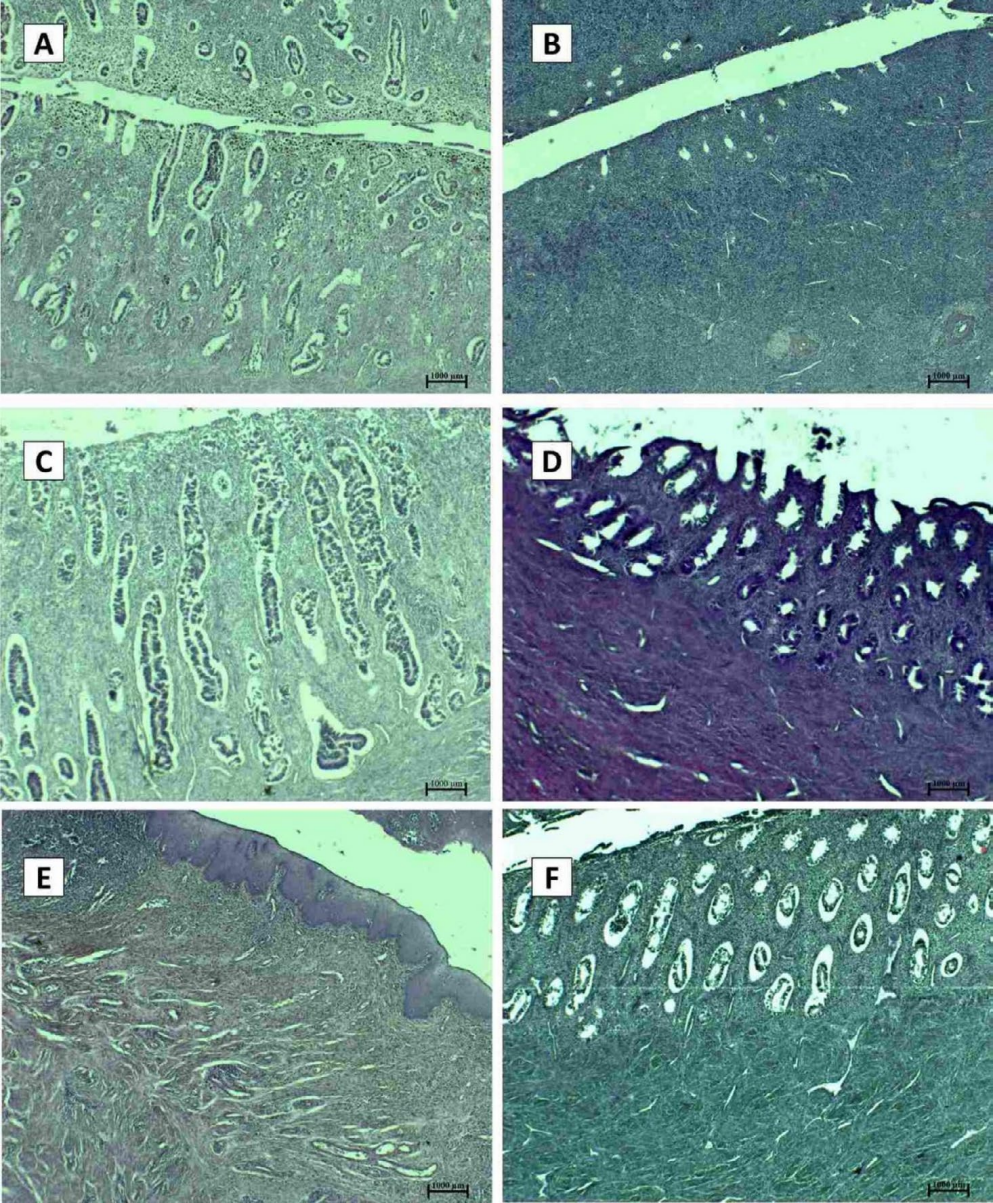
Histological features of the uterus of *Cacajao calvus* (A, C, E) and *Lagothrix lagotricha poeppigii* (B, D, F) across reproductive phases. (A) Proliferative‐phase endometrium showing hemosiderin deposits within the functional layer, indicating previous cyclical stromal remodeling. (B) Early proliferative‐phase endometrium characterized by a thin functional layer and emerging stromal cell proliferation. (C) Secretory‐phase endometrium displaying densely developed, tortuous endometrial glands extending throughout the functional layer. (D) Proliferative‐phase endometrium with numerous rounded, uniformly distributed endometrial glands. (E) Cervical region exhibiting a focal inflammatory infiltrate of polymorphonuclear leukocytes adjacent to the luminal epithelium. (F) Proliferative‐phase endometrium with rounded glands embedded in a moderately cellular stroma. Staining: Hematoxylin–Eosin (H&E), 50× magnification. Images obtained using Nikon trinocular microscope and NIS‐Elements 4.0 software.

Concerning the overall percentage of type III collagen, the early proliferative phase was greater expression in the functional layer with a significative difference when compared to the final proliferative (*p* < 0.001) and secretory phases (*p* < 0.001). In contrast, in the basal layer the percentual of type III collagen was lower in the early proliferative than in the final proliferative (*p* = 0.02), and secretory periods (*p* = 0.006). The histological characteristics of the uterus of *C. calvus* and *L. poeppigii* in the early proliferative, late proliferative, and secretory phases can be observed in Figure 5.

## 4 Discussion

Histological evaluation of endometrial collagen has been documented in several domestic species and in humans; however, to our knowledge, no published study has addressed this topic in non‐menstruating Neotropical primates. Our findings, therefore, provide the first detailed description of the cyclical variations of endometrial collagen in two New World primate species. By demonstrating specific phase patterns in type I and type III collagens throughout the reproductive cycle, this study contributes unprecedented comparative data to the reproductive biology of Neotropical primates and establishes a frame of reference that can support future investigations into uterine physiology and fertility in these taxa.

Regarding endometrial collagen, females of *L. poeppigii* and *C. calvus* showed low expression in the basal layer at the beginning of the proliferative phase, increasing with the advancement of the reproductive cycle. In contrast, collagen expression in the functional layer was higher at the beginning of the proliferative phase and decreased throughout the development of the endometrium, growing again after ovulation. As observed in this species, other non‐menstruating animals undergo cyclic endometrial remodeling through hormonal regulation, exhibiting characteristics similar to those observed in humans [21, 22]. In cattle [23], mice [24], dogs [25], pigs [26], and common marmoset ([27]), cyclic patterns of proliferation and apoptosis similar to those in primates have been described, even in the absence of endometrial shedding. Estrogen and progesterone coordinate structural changes in the endometrium, inducing successive phases of proliferation, differentiation, and apoptosis ([28]), which are accompanied by remodeling of the ECM [29] and angiogenesis [30].

A reduction in collagen in the basal layer is observed due to the regression of the *corpus luteum* and subsequent decrease in progesterone levels in the absence of pregnancy [22]. This hormonal change prepares the uterus for the beginning of a new cycle by decreasing the expression of growth factors and stimulating the synthesis of matrix metalloproteinases (MMPs), responsible for the degradation of collagen in the ECM and rupture of the functional layer [31, 32]. As the proliferative phase progresses, estrogen levels begin to increase from the development of ovarian follicles and stimulate the action of fibroblasts and the production of transforming growth factor beta (TGF‐β), which enhances collagen synthesis [33, 34]. In the functional layer, collagen reduction is observed throughout the proliferative phase, which may occur due to estrogen stimulation of the apoptotic activity of macrophages in the subepithelial stroma to remodel the ECM and allow tissue expansion for gland development [35, 36].

According to this study, the expression of type I collagen was much higher than that observed for type III collagen, like that reported in the literature [6, 37]. The abundant expression of collagen in the endometrium has been reported in histological studies of the reproductive tracts of other Neotropical species, such as the Brown Howler monkey [38] and the Marca's marmoset [39]. However, these studies focused primarily on histological characterization and did not quantitatively evaluate collagen expression across different stages of the reproductive cycle. In women, collagen I is more prevalent in the endometrium throughout the menstrual cycle as well as during pregnancy, while collagen III is present during all phases of the menstrual cycle but less abundant than collagen I during the first trimester of pregnancy [5]. Type I collagen is the most common type of protein in the ECM and is found in connective tissues. It plays a role in fibril formation, which influences the structural support and mechanical strength of tissues, allowing the integrity of the endometrium during the proliferative phase [40, 41]. In addition, it stabilizes the three‐dimensional structure of tissues and assists in cell adhesion, proliferation, angiogenesis, and tissue regeneration [14, 42]. Tissue remodeling is necessary during each phase of the uterine cycle to make the environment receptive to a possible pregnancy through dynamic changes in desquamation, regeneration, and differentiation [43]. For this reason, collagen expression varies throughout the reproductive cycle under the influence of uterine hormones [44].

In *L. poeppigii* and *C. calvus* , a notable difference was observed in the expression of type I collagen in the endometrium. Specifically, there was a reduction in collagen expression within the functional layer during the final proliferative phase. In the basal layer, the lowest collagen expression occurred at the beginning of the proliferative phase, with increased levels during the secretory phase. Studies demonstrate that the endometrium of estrous and menstrual cycle species presents collagen types I and III, whose organization and variation throughout the reproductive cycle play a crucial role in tissue preparation for decidualization and embryonic implantation [6, 45, 46]. In *L. poeppigii* and *C. calvus* , it is observed that type I collagen decreases in the functional layer at the end of the proliferative phase, a period in which the tissue thickens and undergoes intense remodeling to ensure elasticity and accommodate glandular development [47], which influences the stability of reproductive tissues for future placentation [48]. In the basal layer, its expression is lower at the beginning of the proliferative phase and increases in the secretory phase, reflecting the need for structural support and mechanical resistance at this stage [6, 45]. Furthermore, type III collagen has a distinct distribution, with a superficial location and a possible regulatory function in fibrillogenesis, being found mainly in the center of thick fibrils, while type I collagen does not present a specific pattern along the fibrils. Finally, it is noteworthy that, during gestation, the endometrium also exhibits an unusual form of type V collagen, detected only in pregnant endometrium [46], reinforcing that the quantitative and organizational variations of collagen types, especially type I, are determinants for endometrial remodeling and for the proper establishment of decidualization.

During the secretory phase, the *corpus luteum* secretes progesterone, which stimulates the production of TGF‐β and platelet‐derived growth factor (PDGF). These factors play crucial roles in regulating the synthesis of endometrial collagen and promoting fibroblast proliferation [33, 49, 50, 51]. Kaitu'u et al. [52] observed that MMP13, a homolog of MMP1 found in primates, degrades mouse uterine tissue only at the end of the secretory phase. Thus, in the absence of pregnancy, decreased progesterone levels lead to the production of MMPs, which degrade collagen in the ECM of the basal layer. This process prepares the tissue for desquamation by stimulating inflammatory responses [53]. In females undergoing the estrous cycle, the process occurs through epithelial reabsorption with tissue rupture, resulting in fibrin. In contrast, females in the menstrual cycle share a reduction in collagen expression, vascular rupture, and extravasation of red blood cells into the subepithelial region [15].

Type III collagen is often found alongside type I collagen in tissues, and its principal function is to contribute to the structural composition of hollow organs such as the uterus, as well as larger blood vessels [54]. Type III collagen acts in the regulation of tissue regeneration, although its specific role is not understood [14]. Its fibers interact with platelets in the blood coagulation cascade, and it is a relevant signaling molecule during wound healing [55]. Furthermore, this collagen is involved in cell adhesion, migration, proliferation, and differentiation via its interaction with integrins [49]. Increased expression of type III collagen in other organs is typically associated with pathological conditions that promote fibrosis [54].

One possibility for the low identification of type III collagen fibers may be due to the high expression of type I collagen in the endometrium [5, 56]. López De Padilla et al. [9] describe Picrosirius red staining is caused by overstaining by the red chromogen, which may make it hard to assess type III collagen expression. The overlap of type I collagen occurs because its diameter is smaller than that of type I collagen [57], and both types can sometimes appear in the same fibrils with less observation of type III collagen fibers [58, 59]. Although polarized light staining has been used for the qualitative analysis of type I and III collagen fibers in other studies [8, 20, 60], recent publications have raised questions about its effectiveness in accurately distinguishing between the two fiber types. Piérard [61] and Lattouf et al. [56] observed that collagen fibers changed color from red to green when rotated during microscopic evaluation. López De Padilla et al. [9] also noted discrepancies in the differentiation of collagen types when combined with immunohistochemistry as a positive control.

When studying the uterus, Picrosirius red staining associated with polarized light microscopy was valuable in revealing the organization and orientation of collagen fibers. This method can also measure the amount of collagen content during different phases of the uterine cycle, including in normal or pathological tissues. In addition, morphometric analyses were used to study and quantify the remodeling of the collagen network. Evaluation of the uterus of the *L. poeppigii* and *C. calvus* can help in the characterization of the reproductive cycle phase and of the ECM, including collagen morphometry. Future studies with immunohistochemistry are encouraged to evaluate matrix metalloproteinases and changes in endometrial architecture.

## Funding

This work was supported by the Brazilian National Council for Scientific and Technological Development (CNPq), 305821/2017‐2, 4000881/2019‐5, 400800/2019‐5, 316750/2021‐2. Coordenação de Aperfeiçoamento de Pessoal de Nível Superior, PROCAD/Amazônia, 21/2018.

## Conflicts of Interest

The authors declare no conflicts of interest.

## Data Availability

The data that support the findings of this study are available from the corresponding author upon reasonable request.
